# MRChem Multiresolution Analysis Code for Molecular Electronic Structure Calculations: Performance and Scaling Properties

**DOI:** 10.1021/acs.jctc.2c00982

**Published:** 2022-11-21

**Authors:** Peter Wind, Magnar Bjørgve, Anders Brakestad, Gabriel A. Gerez S., Stig Rune Jensen, Roberto Di Remigio Eikås, Luca Frediani

**Affiliations:** † Department of Chemistry, UiT The Arctic University of Norway, N-9037Tromsø, Norway; ‡ Algorithmiq Ltd., Kanavakatu 3C, FI-00160Helsinki, Finland

## Abstract

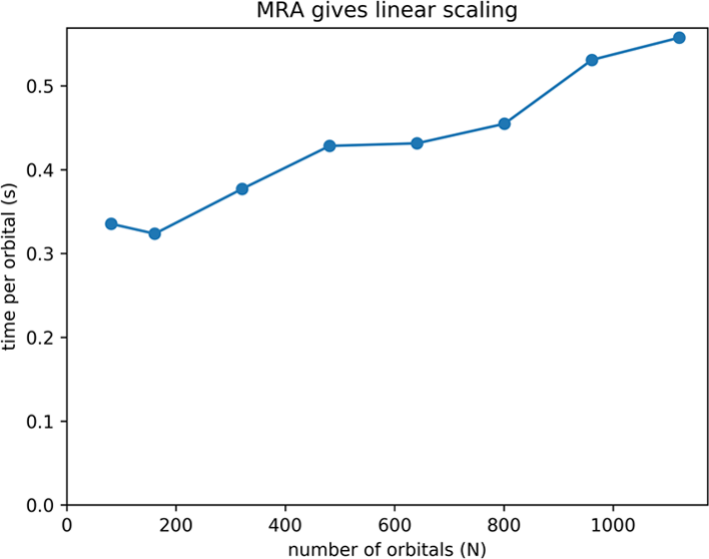

MRChem is a code for molecular electronic structure calculations, based on a multiwavelet adaptive basis representation. We provide a description of our implementation strategy and several benchmark calculations. Systems comprising more than a thousand orbitals are investigated at the Hartree–Fock level of theory, with an emphasis on scaling properties. With our design, terms that formally scale quadratically with the system size in effect have a better scaling because of the implicit screening introduced by the inherent adaptivity of the method: all operations are performed to the requested precision, which serves the dual purpose of minimizing the computational cost and controlling the final error precisely. Comparisons with traditional Gaussian-type orbitals-based software show that MRChem can be competitive with respect to performance.

## 1 Introduction

Gaussian-type orbitals (GTOs) and more generally linear combination of atomic orbitals (LCAOs)^1^ are well established as a standard for ab initio molecular electronic structure calculations. As their shape is closely related to the electronic structure of atoms, even very small basis sets with only a few functions per molecular orbital (MO) can give reasonable results for describing molecular properties. However, for extended systems, the description of each orbital still requires contributions from the entire basis to ensure orthogonality. Without further precautions, even when using localized orbitals, a large proportion of the coefficients will be very small for those systems.

In a multiresolution analysis (MRA) framework like multiwavelets (MWs), the basis can adapt according to the function described (for an in-depth review of the multiresolution analysis (MRA) method in the field of quantum chemistry, see ref (2)). The available basis is in principle infinite and complete, and, in practice, it is dynamically adapted to the local shape of the function and the required precision. This can require the real-space basis to comprise millions of elementary functions for each orbital. In this sense, the method starts with a big disadvantage compared to LCAO basis sets. On the other hand, the exponential growth of available computational resources has in recent years enabled multiresolution analysis (MRA) calculations on systems comprising thousands of atoms.^3,4^

Two main challenges need to be addressed to achieve adequate performance: the large number of operations to perform and the large memory footprint. The former is addressed by limiting the numerical operations to those that are strictly necessary to achieve the requested precision: rigorous error control at each elementary operation is the main strength of a MW approach, enabling effective screening. The latter is achieved by algorithmic design: beyond a certain system size, not all data can be stored in local (i.e., fast access) memory. On modern computers, data access is generally more time-consuming than mathematical operations, especially if the data is not available locally.^1^ The computer implementation must be able to copy large amounts of data efficiently between compute nodes and the algorithm must be devised to reuse the data already available on the compute node or in cache memory when possible. In this article, we will present the main implementation features of our multiresolution analysis (MRA) code, MRChem,^5^ to tackle those challenges, thus enabling calculations on large systems at the HF level.

Beyond the effective thresholding that screens numerically negligible operations, the large computational cost is addressed by parallelization either at the orbital level or through real-space partitioning. This dual strategy allows us to minimize the relative cost of communication and the overall computational costs. Further, the most intensive mathematical operations are expressed as matrix multiplications, which allows for optimal efficiency. Using localized orbitals, the adaptivity of the MW description will significantly reduce the computational effort required to compute the terms involving remote orbitals. Within a multiresolution analysis (MRA) framework, the operators will exhibit intrinsic sparsity, even if the orbitals have contributions from remote regions. This is achieved because the different length scales are naturally separated through the adaptive grid representation. This opens the way for a method that scales linearly with system size (*N*), where the linearity arises naturally from the methodology, rather than being obtained with ad hoc techniques, such as fast-multipole methods^6^ for Fock matrix construction, or purification of the density matrix.^7^

The large memory requirement is addressed by storing the data in a distributed “memory bank”, where orbitals can be sent and received independently by any CPU. The total size of the memory bank is then only limited by the overall memory available on the entire computer cluster. Benchmark calculations at the HF level show that MRChem is able to describe systems with thousands of electrons. The implementation exhibits near-linear scaling properties, and it can also be competitive with state-of-the-art electronic structure software based on LCAO methods.

## 2 Solving the Hartree–Fock Equations with Multiwavelets

We consider the self-consistent field (SCF) equations of the HF method
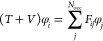
1where the Fock matrix elements *F*_*ij*_ are obtained as
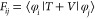
2In the above equations,  is the kinetic energy operator, and the potential *V* contains the nuclear–electron attraction *V*_nuc_, the Coulomb repulsion *J*, and the exact exchange *K*. To solve the self-consistent field (SCF) equations within a MW framework, we rewrite the differential eq (1) as an integral equation
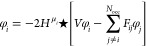
3where * stands for the convolution product. This form is obtained by recalling that the shifted kinetic energy operator *T* – ϵ has a closed-form Green’s function^8,9^
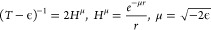
4By setting ϵ_*i*_ = *F*_*ii*_ and , the diagonal term is excluded from the summation in eq (3). It can be shown that iterating on the integral equation corresponds to a preconditioned steepest descent. Practical applications show that this approach is comparable in efficiency (as measured in the rate of convergence of the self-consistent field (SCF) iterations) to more traditional methods.^10^

A MW representation does not have a fixed basis. It is therefore not possible to express functions and operators as vectors and matrices of a predefined size, and the virtual space is to be regarded as infinite. Still, each function has a finite, truncated representation defined by the chosen precision threshold, but this representation will in general be different for different functions. It is therefore necessary to develop working equations that allow the direct optimization of orbitals. On the other hand, only occupied orbitals are constructed and the coupling through the Fock matrix in eqs (1) and (3) is therefore limited in size. Another advantage is that, within the requested precision, the result can be formally considered exact, and exploiting formal results valid in a complete basis—most notably the Hellmann–Feynman theorem—becomes straightforward.^11^

Differential operators such as the Laplacian pose a fundamental problem for function representations that are not continuous, and a naive implementation leads to numerical instabilities. Robust approximations are nowadays available,^12^ but for repeated iterations avoiding the use of the Laplacian operator is still an advantage. This is the main reason for using the integral form in eq (4) rather than the differential form.

## 3 Implementation Details and Parallelization Strategy

MW calculations can be computationally demanding. The total amount of elementary operations, the large amount of memory required, and the capacity of the network are important bottlenecks.

In practice, a supercomputer is required for calculations on large systems. For example, the representation of one orbital can demand between 10 and 500 MB of data, depending on the kind of orbital and the precision requested (see Section 4.2.1 for details). Moreover, several sets of functions are necessary in a single self-consistent field (SCF) iteration (orbitals at the previous iterations, right-hand side of equations, etc.). Large systems will eventually require more memory than is locally available on a single compute node in a cluster, and distributed data storage becomes mandatory. At the same time, the self-consistent field (SCF) equations will require pairs of orbitals, i.e., all of the data must at some point be accessible locally.

For an efficient solution scheme on a cluster architecture, the problem needs to be partitioned into smaller portions that can be computed with as little transfer of data as possible between the different compute nodes of the cluster.

### 3.1 Localized Versus Canonical Orbitals

Although localized and canonical orbitals represent the same *N*-electron wave function, the numerical operations required to solve the equations will have different scaling properties in a canonical or localized representation. Because of the inherent adaptivity of the MW approach, multiplying two orbitals, or applying an operator onto an orbital can be done more efficiently if the orbitals are localized in space. Even if the orbitals have non-negligible values in a large portion of space, the adaptivity properties will make sure that the calculations in the parts where the orbitals are small are done at a coarser level of accuracy, and therefore faster than in the regions with larger values.

We use the Foster–Boys localization criterion.^13^ The unitary, localizing orbital rotation is computed by minimizing the sum of the occupied orbitals’ second central moment.^14^ See also Sections 3.2 and 3.5 for more details on the practical implementation.

### 3.2 Local (Real-Space) vs Orbital Representation

**Figure 1:**
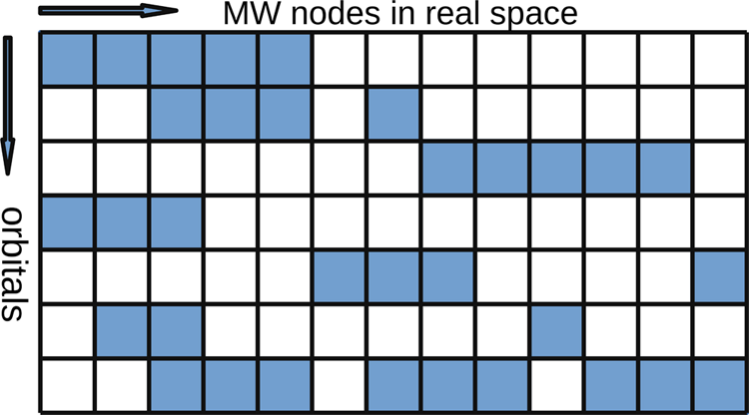
Real-space vs orbital representation. A dark cell represents a MW node occupied by the corresponding orbital. In an orbital representation (rows), the data from each row is stored and retrieved together. In a local representation (columns), the data from each column is stored and used together. The most convenient representation depends on the operation at hand and it is therefore best to be able to switch from one to the other.

Two main strategies can be adopted to distribute the memory storage requirements for orbitals throughout the cluster. The first strategy follows verbatim the mathematical expressions: the HF equations are written in terms of orbitals, and therefore it is natural to keep the data representing each orbital as a separate C++ structure. The second strategy is based on real-space partitioning: contributions from all the orbitals in a given portion of real space are stored together, i.e., as a separate C++ structure that is computationally cheap to retrieve (see Figure 1).

Both strategies have advantages and disadvantages, and in practice, it is convenient to switch between them depending on the given operation.

Taking as an example the computation of the Fock matrix elements in eq (2), we will show why it is advantageous to shift to a real-space partitioning approach.^2^ Operator matrix elements are evaluated as
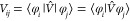
5where φ*_i_* and φ*_j_* are occupied orbitals and *V̂* is any one-electron operator, such as the nuclear potential operator. If the application of the operator (|*V̂*φ_*j*_⟩, ∀*j*) is considered as an *O*(*N*_occ_) operation, computing the entire matrix scales formally as *O*(*N*_occ_^2^).

Both the φ*_i_* and the result of the operator application τ_*j*_ = *V̂*φ_*j*_ are represented either through the compressed or reconstructed representation, as described in Section 1 in the Supporting Information. For instance, the compressed representations for φ*_i_* and τ_*i*_ would yield, respectively
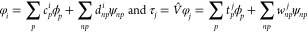
6where *n* runs over all of the included MW nodes (see the Supporting Information (SI) for details about representing functions using MW).

When representing functions in 3 dimensions, using a tensor-product basis and localized orbitals, both *p* and the number of MW nodes, are typically of size ∼ 10^4^. To perform a scalar product, it is sufficient to recall that the scaling/wavelet basis is fully orthonormal
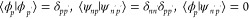
7which then yields the following result for an operator matrix element between two orbitals
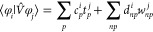
8where now the sum over the MW nodes *n* only extends to those which are present for both functions.

Ignoring for simplicity the scaling contribution which has negligible impact on the performance, this operation is equivalent to a matrix multiplication *D* × *W*^*T*^ with *D*_*ia*_ = *d*_*np*_^*i*^ and *W*_*ja*_ = *w*_*np*_^*j*^, and *a* is the compound index *a* = *np*. However, for large systems, it is not possible to hold the full matrices in local memory. The molecular orbital (MO)-like approach is then to sum over all compound indices *a* for each pair *i*,*j* independently. In this way, each pair of coefficients *d*_*np*_^*i*^ and *w*_*np*_^*k*^ is used only once after each memory fetch. On modern computers, memory access is much more time-consuming than the mathematical operations themselves, up to a factor of 100 for most CPU-based systems. A faster implementation is to consider a specific MW node *n* and perform the product for all *i*, *j*, and *p* values within this node
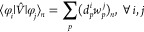
9this results in a series of independent matrix multiplications, thus fully utilizing the capabilities of the computer.

To perform the operation in this way, the storage of the orbitals in memory must be adapted. Instead of storing each orbital separately (orbital or MO representation), the values of all of the orbitals at specific positions in space (i.e., a MW node) must be easily accessible (local representation: all of the data covering a specific MW node is stored on the same compute node, as an independent C++ structure). Switching from an orbital to a local representation is an *O*(*N*) operation, where *N* measures the system size, typically the number of orbitals. If all *i* and *j* are included, the number of operations still scales formally as the square of the number of orbitals. However, for a given MW node *n*, the products only need to be explicitly performed if both functions (indices *i* and *j*) have a non-negligible contribution to the MW node *n*. When orbitals are localized, the number of such contributions will eventually scale linearly with the system size.

Linear combinations of orbitals can be addressed similarly. Let us consider the linear combination
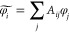
10where *A*_*ij*_ is the coefficient matrix of the transformation. In a linear transformation, each MW node *n* can be transformed independently, to obtain the corresponding coefficients for φ̃_*i*_
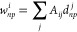
11Here also, the number of localized orbitals contributing to a specific MW node *n* grows as *O*(1) with system size. When constructing φ̃_*i*_, the grid is successively refined so that only values corresponding to MW nodes with wavelet norms above the required threshold are actually computed. Moreover, due to linearity, this operation is carried out both for scaling and wavelet coefficients, thus avoiding the need to perform any MW transforms.

### 3.3 Adaptivity and Precision Thresholding

The most powerful idea of multiresolution analysis (MRA) is to limit the precision of intermediate operations to what is strictly necessary to achieve the requested precision in the final result. This guiding principle can be exploited at several levels

1. Elementary mathematical operations on a function in a multiwavelet representation will automatically refine the local basis (i.e., grid) of the resulting function, but only until the required precision is achieved. The computational effort required will vary according to the position and shape of the functions or operators. For example, the product of two functions that are localized in different parts of space will normally be done faster than the square of each of them.
2. Only those parts of functions with absolute value (norm of the MW node) above a defined threshold will be retrieved before even processing them; this is used e.g., in the scheme presented in Section 3.2.
3. A self-consistent field (SCF) calculation can be initiated at low precision and the final result can be used as starting guess for a calculation with higher precision. In contrast to finite AO basis sets, which are usually not subsets of each other, a multiwavelet function computed at a given precision, can be directly used as a valid representation at a different precision.

### 3.4 Exchange Screening

The exchange terms require the evaluation of
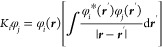
12for all  orbital pairs *i*,*j*.

The evaluation of eq (12) can be divided into three operations

- Product of φ_i_^*^(***r***′) and φ_*j*_(***r***′)
- Application of the Poisson operator,
- Product with φ_*i*_(***r***),

The application of the Poisson operator gives a long-range potential that decays as . However, if the orbital φ_*i*_(***r***) is localized, only the part of the potential where φ_*i*_(***r***) is large enough needs to be computed. More precisely, the potential needs only to be computed up to a precision that is inversely proportional to the value of φ_*i*_(***r***) at that point. The Poisson operator can therefore use a locally defined threshold, where the required precision of the operator application is determined using local information from a proxy for the result function.

As for all MW representations of functions, the output of a Poisson operator application uses an adaptive grid: when a new MW node is computed, the norm of the wavelet part is compared to the precision threshold to decide whether to further refine the representation or not. In general, the precision threshold is fixed: it is one and the same over the entire space. For the HF exchange, we already know that the result of convolution with the Poisson kernel will only be used as part of the product with φ_*i*_(***r***). Thus, the precision threshold can be multiplied by the local value of the norm of φ_*i*_(***r***) (the reference function). This procedure will yield a grid that is adapted to the final result, with increased precision corresponding to the regions with non-negligible values of φ_*i*_(***r***).

Several reference functions can be provided, and the precision at a given MW node is determined by the largest value of the norm of those functions at the node. This allows us to treat the two terms  and  using the same potential . Also complex functions can take advantage of this feature since the real and imaginary parts of φ_*i*_(***r***) can then use the same potential.

Without screening, the number of exchange terms grows as *N*_occ_^2^ and each term requires the application of the Poisson operator. Distant orbitals that do not overlap, do not contribute, and therefore proper handling of the screening will be reflected in a cost of the exchange terms that grows linearly in size for large enough systems (if the required overall absolute precision is held constant). Additionally, if the product of φ_i_^*^(***r***′) and φ_*j*_(***r***′) is small enough, the calculation of the corresponding term can be avoided altogether. As shown in Section 4.4 this leads to an effective long-range screening.

### 3.5 *O*(*N*_occ_^3^) Terms

In our implementation, there are two steps with an operation count that formally scales with the third power of the number of (occupied) orbitals: the localization and the orthogonalization steps. The former involves the product of matrices with sizes *N*_occ_ × *N*_occ_; the latter performs the diagonalization of a Hermitian matrix and subsequent matrix multiplications, both with cubic formal scaling.

Matrix operations can be performed extremely efficiently on any computer using standard linear algebra libraries, and those terms will not contribute significantly to the total computation time before considering sizes larger than roughly *N*_occ_ = 1000 for serial matrix multiplications, or 10^4^–10^5^ for parallel, distributed matrix multiplications (not yet implemented). We have therefore not yet rendered such operations faster than their formal scaling.

We note that both steps are not specific to the multiwavelet approach. As a matter of fact, LCAO approaches may require those steps to be performed on the virtual orbitals too. For large systems, it might also be possible to exploit the numerical sparsity of the computed matrices.

### 3.6 Parallelization

MRChem is parallelized using the message passing interface (MPI) standard^15−17^ and OpenMP threading.^18,19^ The MPI processes are partitioned into three groups, which will be described in the following section.

#### 3.6.1 MPI “Worker” Processes

They perform mathematical operations. These processes have also access to multiple threads for OpenMP parallelization. The large majority of the cores are assigned to worker processes.

The low-level operations (on one or two orbitals or MW functions, and on MW nodes) are included in the MRCPP library. MRCPP will perform tree operations as a set of operations on MW nodes. Those operations will then be performed in parallel by OpenMP threads. The thread parallelism is transparent for the library user, who will only need to set the number of OpenMP threads.

#### 3.6.2 Memory Bank Processes

These processes are only receiving and distributing data to the workers, they do not perform major mathematical operations and are not threaded. Bank processes will impose a high load on the network; it is therefore important to distribute them on all compute nodes to avoid saturation of the local network. Since the memory bank processes are different from the worker processes, the worker processes can access all of the data without direct communication with other workers (in effect, one-sided memory access). Efficient transfer of data is the key to a successful parallelization scheme.

In the MW approach, orbitals are represented as tree structures. The root nodes of the MW tree cover the entire space of the system, and each subdivision defines branches. To facilitate the transfer of those functions, the tree structure and the MW node data are stored in piecewise contiguous memory. This allows the data to be sent efficiently among MPI processes: only the position in memory of the start and end of the pieces are required, alongside some metadata. A MW tree can then be transferred without intermediate buffering and with little overhead. The MRCPP library is able to transfer MW trees (or orbitals) between message passing interface (MPI) processes easily and efficiently.

#### 3.6.3 Task Manager

In a localized orbital approach, given the matrix representation ***V*** of an operator *V̂*, many of its matrix elements *V*_*ij*_ = ⟨φ_*i*_|*V̂*φ_*j*_⟩ will be small. The multiresolution analysis (MRA) framework implemented in the MRCPP library is inherently adaptive and small elements are computed faster. To exploit such a feature, it is important to equip the library with a dynamic distribution of tasks. In practice, the matrix ***V*** is partitioned into blocks, each block representing one task to be performed by a worker. In a local representation, each MW node is simply assigned to a separate task. When a worker is done with one task, it can ask the task manager for a new task until all tasks are completed.

One single MPI process is reserved for managing the task queue. The only purpose of this process is to keep account of the tasks that have been assigned and the tasks left, and distribute unsolved tasks to the workers. It does only send and receive small amounts of data at a time (a few bytes) and will not be saturated with requests.

## 4 Benchmark Results

In this section, we will show how MRChem performs for some simple systems of different sizes. MRChem is still under development and the results shown here are just a snapshot of the present situation. The goal is not to determine the limitations of the model, but rather to show that it behaves as intended and that the development is going in the right direction.

All benchmark runs presented here were performed on “Betzy”, a Bull Sequana XH2000 computer cluster, with 128 cores and 256 GiB memory per compute node, and InfiniBand HDR 100 interconnect.^20^

All timings given in this section are subject to a degree of uncertainty. There are multiple reasons for this. The runs have been executed in an MPI parallel framework on a supercomputer shared with other users. Several algorithms exploit a dynamic allocation of tasks; as a consequence, the same calculations can have different workload distributions in two repeated identical runs. The network capacity may also be affected by other users. Finally, the time spent in different parts of the code may not be well defined: in a parallel framework, the same code will start and end at different times for different processes. In principle, the time for one block should include the time for all processes to be finished; however, in practice, processes are allowed to continue without waiting, until they need data that is not yet available. The time for a similar self-consistent field (SCF) cycle may vary by up to 10%. However, this accuracy should be sufficient and reflect the main trends faithfully.

### 4.1 Scaling with System Size

**Figure 2:**
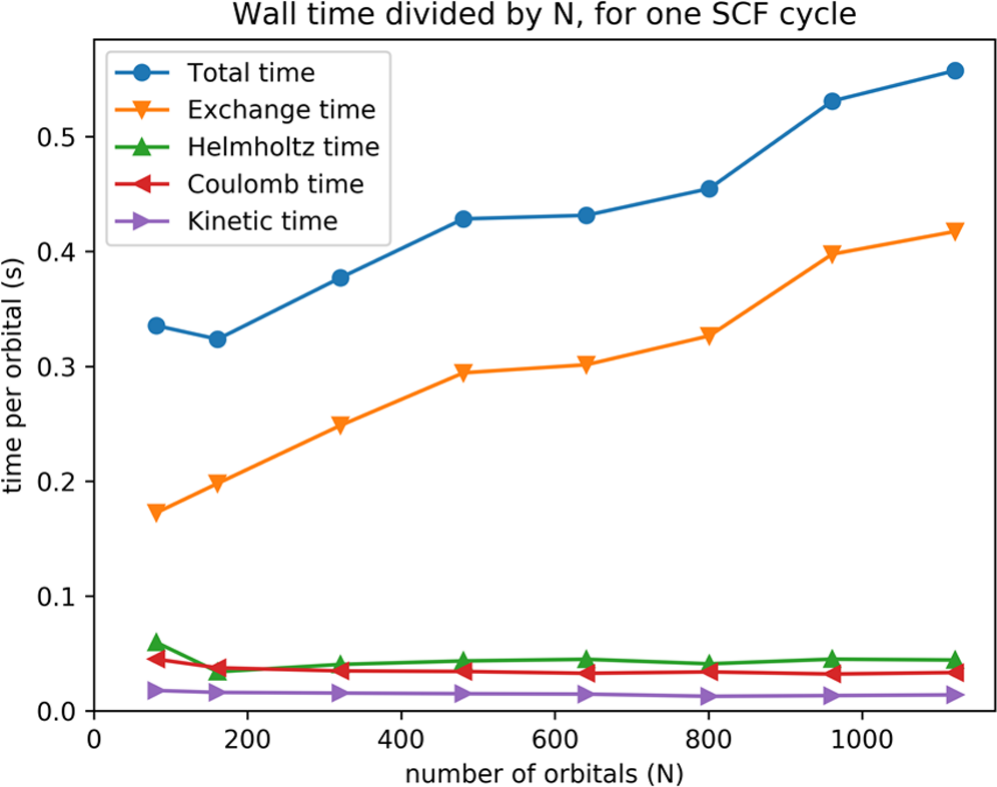
Timings divided by number of orbitals for one self-consistent field (SCF) cycle for the linear alkanes C_*n*_H_2*n*+2_ (*n* = 20, 40, 80, 120, 160, 200, 240, and 280). All calculations were performed using eight compute nodes. Total time refers to the time spent in one Hartree–Fock self-consistent field (SCF) cycle, when it is close to convergence. Exchange time refers to the time spent computing the exchange contributions. Coulomb time refers to the time used for computing the Coulomb potential of each orbital, summing them up, and distributing the total potential to all of the MPI processes. Kinetic time refers to the time spent computing the kinetic energy of each orbital. Helmholtz time is the time for constructing and applying the Helmholtz operator. Constant time indicates linear scaling.

Figure 2 shows computation times divided by the number of orbitals for alkanes of different lengths. For a linear scaling method, the time per orbital should be approximately constant. The time per orbital less than doubles going from the smallest (81 orbitals) to the largest (1121 orbitals) alkane. This shows that the scaling with system size is mostly linear, with a small nonlinear contribution.

The application of the Exchange operator is by far the most time-consuming part of the self-consistent field (SCF) process. The other parts are showing close to linear scaling behavior. The number of non-negligible exchange contributions should ideally grow linearly with system size for large enough systems, and therefore the exchange computation time per orbital should be constant. However, exchange contributions are the result of a sum of terms, and if the number of terms increases, the accuracy of each individual term must be increased to achieve a fixed absolute precision in the final result. In our implementation, this is taken care of by increasing the required precision of the individual terms by a factor proportional to the square root of the number of orbitals. This is the main reason why our run times are exhibiting a slight nonlinearity.

### 4.2 Scaling with Precision

Table 1 shows the results of calculation carried out with different precisions on two molecules, valinomycine (C_54_H_90_N_6_O_18_, 300 orbitals) and the gramicidin dimer (C_198_H_276_N_40_O_34_, 1008 orbitals). MW5 (10^–5^), MW6 (10^–6^), and MW7 (10^–7^) denote the value of the user-defined precision parameter. Our examples show a factor of 2.5 increase in computing time for each 10-fold increase in precision (one additional digit). A similar test with Gaussian-type orbitals (GTOs), performed increasing the basis set size, shows a much less favorable scaling: see examples in Table 5.

**Table 1:** Time (in Seconds) for Different Terms in the Self-Consistent Field (SCF) Cycle^a^

		exchange	Coulomb	kinetic energy	Helmholtz	total
valinomycine	MW5	71.4	9.2	2.7	7.4	97.4
	MW6	182.4	25.3	6.7	21.3	251.2
	MW7	442.8	78.8	17.1	66.4	644.4
gramicidin	MW5	115.2	18.4	4.2	8.0	163.7
	MW6	312.0	45.8	14.6	25.5	428.9
	MW7	813.6	127.8	28.5	83.1	1196.9

a 16 and 64 compute nodes used for valinomycine (C_54_H_90_N_6_O_18_, 300 orbitals) and gramicidin dimer (C_198_H_276_N_40_O_34_, 1008 orbitals), respectively. Total timing is dominated by the HF exchange. The additional cost for each precision increase is roughly a factor of 2.5–3 for all contributions.

#### 4.2.1 Size of Orbital Representation

In a localized approach, the orbital functions have negligible values in remote regions of space. In an LCAO approach (if no special filtering is applied) the representation of an orbital will span the entire represented space since the basis will increase with the system size. In the multiresolution analysis (MRA) approach orbitals will essentially be represented using locally defined functions and their size will depend only weakly on the size of the entire system. This is confirmed directly in our calculations, see Table 2. Those results were obtained using a threshold parameter of 10^–5^ (MW5), 10^–6^ (MW6), and 10^–7^ (MW7). The individual size of the orbitals will of course vary greatly according to their type; for example, core orbitals are simpler and therefore require less data at a given precision, more diffuse functions have more features, and their representation requires more data. In the molecules presented here, the largest orbital may be 2–4 times larger than the average, and the smallest a third of the average size. Our empirical observations show a factor in the range of 2–2.2 increase in orbital size for each ten-fold increase in precision. An increase in the number of coefficients describing the orbitals will clearly increase the computational cost of each operation using this orbital. In addition, an increase in precision will also extend the range of orbitals with non-negligible interactions, i.e., demands less aggressive screening. The number of orbitals combined with the average size of the orbitals can be taken as an indicator of the amount of computational resources (memory, CPU time, and network) required to solve the equations.

**Table 2:** Average Orbital Size for Different Molecules in Megabytes (MB) for Increasingly Tighter Precision Parameters^a^

	C_20_H_42_	C_40_H_82_	C_80_H_162_	C_160_H_322_	valinomycine	gramicidin
MW5 orbital size (MB)	35	43	43	46	49	55
MW6 orbital size (MB)	80	85	90	92	115	136
MW7 orbital size (MB)	158	167	176	180	245	306

a The average orbital size is evaluated in terms of the number of MW nodes or coefficients required. The largest molecule in the alkane series is almost independent of the molecule size.

### 4.3 Scaling with Number of Compute Nodes

**Figure 3:**
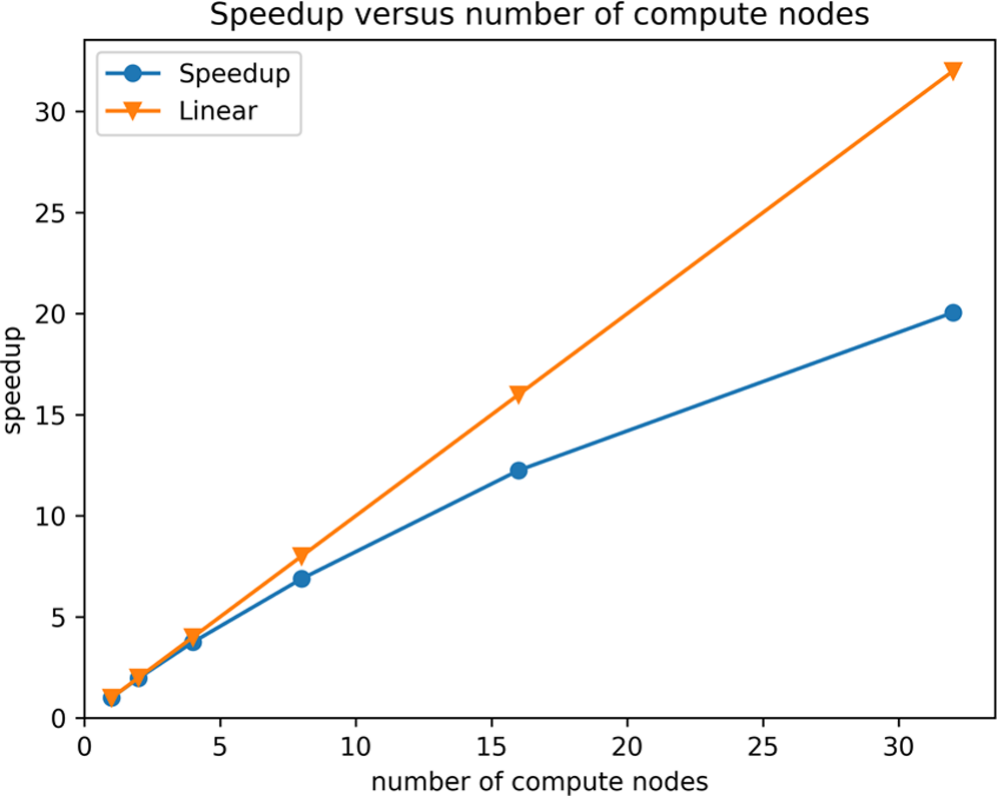
Speedup is the inverse of the time for one Hartree–Fock cycle for the valinomycine molecule (C_54_H_90_N_6_O_18_) at MW4 precision, relative to the time used on one compute node. Each compute node hosts 12 MPI processes, four of which are task manager/memory banks (single-threaded) and eight are worker processes (15 threads each).

The time to perform a full calculation will normally decrease when using more computational resources (compute nodes). Ideally a doubling of the number of computational resources would half the calculation time. However, a larger number of compute nodes implies that an increasing fraction of time must be spent distributing the data around. We define the speedup as the time for a calculation on one compute node divided by the time using *N*_nodes_. Ideally the speedup is equal to the number of compute nodes (linear). In Figure 3, we show the speedup for the valinomycine molecule at MW4 precision for *N*_nodes_ = 1, 2, 4, 8, 16, and 32.

For 32 compute nodes, the time for one SCF cycle is 20 times faster than using 1 compute node, showing a relative efficiency of 20/32 ≃ 63%. For larger systems or higher precision, the efficiency is expected to increase.

### 4.4 Implicit Screening

It is instructive to see more in detail how the adaptivity of multiresolution analysis (MRA) leads to a significant reduction of the computational cost. We will consider linear alkanes of different sizes, as they are easier to compare.

As a first illustration, we will consider the evaluation of the Fock matrix, eq (2) using the formulation from eq (9)
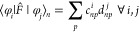
13where *i* and *j* run over the occupied orbitals, *n* runs over all MW nodes (*n* collects all values of *s* and *l* present in the tree) and *p* runs over the MW basis within that node, i.e., three-dimensional polynomials. In the calculations presented in this paper, polynomials up to 7th order in each direction are used, leading to a total of 4096 three-dimensional polynomials (wavelet and scaling) for each MW node. We underline that this choice might be suboptimal for high-precision calculations (MW7), but we have decided to keep the parameter fixed for ease of comparison.

In theory, the MW basis comprises all polynomials at the locally finest scale, in the union of all of the orbital grids. This basis can be very large, but we never explicitly construct it in full. In Table 3, we show the total number of MW nodes present in this basis (i.e., number of indices *n*). As expected, this number grows linearly with the size of the system.

**Table 3:** Number of Terms and Timings for the Fock Matrix Calculation for C_*n*_H_2*n*+2_^a^

*n*	MW nodes	blocks	computed	neglected (%)	fetch (ms)	multiply (ms)
20	6472	42M	1.6M	96.33	164	19
40	12 936	335M	3.8M	98.86	340	38
80	26 120	2691M	10.5M	99.61	532	97
160	52 136	21 422M	30.8M	99.85	981	270

a Precision MW4. The number of fully computed terms increases faster than linearly, but the fraction of time to perform the corresponding multiplications is small.

Let us now define a block as one set of {*i*,*j*,*n*} indices. The total number of blocks is the number of MW nodes *n* multiplied by the square of the number of orbitals. For each block, eq (9) defines the multiplication of the corresponding coefficients for all of the polynomials defined within a MW node. The total number of blocks increases with the third power of the system size. However, if a given MW node is present for one orbital *i*, but not for orbital *j*, it will not contribute to the corresponding matrix element and can be omitted. This follows directly from the orthogonality of the underlying wavelet basis, eq (7). For localized orbitals, *i* and *j* will contribute only to limited subsets of nodes and a large proportion of terms are either zero or have negligible numerical values in relation to the requested precision. They can therefore be discarded without being evaluated. This is a direct consequence of the disjoint support of the MW basis, combined with localization.

As shown in Table 3, the number of non-negligible terms is still increasing faster than linear. This is not necessarily synonymous with computation time increasing faster than linear. To perform the calculations, the code must first fetch the relevant data in the bank, i.e., the matrices *c*_*np*_^*i*^ and *d*_*np*_^*j*^ for given *n*. Then the two matrices are multiplied. As shown in the table the total time used to fetch the data grows slightly slower than linearly. For large systems, the total time to perform the matrix multiplication is proportional to the number of computed terms and grows faster than linear. On the other hand, the matrix multiplication part is done so efficiently that it takes only a small fraction of the total time. In fact, in our implementation, we did not even use threaded matrix multiplication (yet); for large systems, an order of magnitude could be gained on the timings for the matrix multiplication part using threaded matrix multiplication.

Table 4 shows the total number of exchange terms and how many of them are actually fully computed. Since the terms are computed in pairs and the number of non-diagonal pairs is *N*(*N* – 1)/2, their number scales quadratically. The computation of the exchange terms starts with the product of two orbitals. If the product has a norm smaller than a threshold, it can be neglected without having to apply the Poisson operator (which is the computationally expensive part). We see that a large fraction of terms is effectively neglected, and the number of terms remaining scales linearly.

**Table 4:** Number of Terms in the Exchange Calculation for the C_*n*_H_2*n*+2_ Series^a^

*n*	number of orbitals	non-diagonal terms	fully computed	fraction neglected
20	81	3240	1146	65%
40	161	12 880	2548	80%
80	321	51 360	5194	90%
160	641	205 120	10 404	95%

a Precision MW4. The number of terms fully computed becomes proportional to the number of orbitals.

### 4.5 Comparison with ORCA and LSDalton

Table 5 shows the computation time for one self-consistent field (SCF) cycle for the valinomycine molecule computed at the HF level. The accuracy of the total energy and atomization energies are compared with the ORCA and LSDalton results using a pc-1, pc-2, and pc-3 basis sets.^21,22^ Calculations with the pc-4 basis either did not run or did not converge.

**Table 5:** Precision and Performance Comparison with ORCA and LSDalton, for One Self-Consistent Field (SCF) Cycle for the Valinomycine Molecule (C_54_H_90_N_6_O_18_)^a^

program	basis	*N*_P_(*N*_C_)	*E* [a.u.]	error	Δ*E* [a.u.]	error	time [s]
LSDalton	pc-1	2502 (1542)	–3770.20554	2.6e-00	–19.12076	1.1e-01	101
	pc-2	5040 (3600)	–3772.56656	3.0e-01	–19.27598	–4.0e-02	1688
	pc-3	9972 (8052)	–3772.83312	3.3e-02	–19.24894	–1.4e-02	26319
	pc-3^b^	9972 (8052)	–3772.82592	4.0e-02	–19.26353	–2.9e-02	1924
ORCA	pc-12502	2502 (1542)	–3770.19956	2.6e-00	–19.11479	1.2e-01	25
	pc-2	5040 (3600)	–3772.56922	3.0e-01	–19.27865	–4.4e-02	265
	pc-3	9972 (8052)	–3772.83357	3.2e-02	–19.24940	–1.4e-02	3933
MRChem	MW4		–3772.85028	1.6e-02	–19.21937	1.5e-02	117
	MW5		–3772.86560	2.5e-04	–19.23469	2.5e-04	390*
	MW6		–3772.86584	1.0e-05	–19.23493	1.0e-05	1005*
	MW7		–3772.86585		–19.23494		2577*

a *E* is the total energy and Δ*E* is the atomization energy. The error is defined as the difference with the MW7 results. Wall time per self-consistent field (SCF) cycle is reported. *N*_P_(*N*_C_) is the number of GTO primitive (contracted) functions. All computations were done on four compute nodes (i.e., 512 cores), except for MRChem MW5, MW6, and MW7, which were performed using 16 compute nodes (timings are marked with *). Their timings have been multiplied by a factor of 4 for ease of comparison.

b Performed using density fitting (df-def2 basis: *N*_P_ = 9366, *N*_C_ = 7518) and ADMM (admm-3 basis: *N*_P_ = 4560, *N*_C_ = 3768) to accelerate the construction of *J* and *K*, respectively.^23^

We notice that at the lowest precision (MW4), the results have the same quality as the pc-3 basis, but at a fraction of the computational cost. MW calculations show also a rapid convergence pattern with roughly a 2.5 times increase in computational time for each additional digit in the precision. GTO bases show instead a much slower convergence trend: a three-fold error reduction has a cost of more than an order of magnitude. Run times (and possibly the pc-4 convergence) for ORCA^24^ and LSDalton^25^ could likely be improved by tuning different input parameters. We did not attempt to further optimize all of these settings: we are confident that the main picture would not change.

We stress also that it would not be possible to assess the error of GTO calculations, were the precise MW results not available. Reducing the error by increasing the basis set size is computationally demanding if at all possible for large systems. The ability to better control precision is a definitive advantage of the multiresolution analysis (MRA) method: the precision is determined by a single parameter that is independent of the property of interest and that can be chosen on a continuous scale.

## 5 Discussion

We have presented a fully functional implementation of the multiwavelet method and demonstrated that it is capable of handling systems with thousands of electrons at the HF level. The methodology is—almost—linear scaling with system size. The precision can be adjusted as a single, user-defined input variable, which is more intuitive compared to the choice of GTO basis set in traditional methods. For high precision or large systems, the method is competitive with LCAO methods. It is only recently that computers large enough to treat such systems at an affordable computational cost have been available; this may explain why multiresolution analysis (MRA) methods have received relatively less attention so far.

There are certainly many alternative ways to approach the problem and we still expect that significant improvements in the efficiency of the algorithm will be implemented in the coming years: especially the large memory footprint is a serious bottleneck that should be further addressed.

The actual basis used in MRChem can be several orders of magnitudes larger than what is used in large GTO basis. Nevertheless, the computation times are still affordable and even competitive. We believe this is due to modern computers being extremely efficient at performing a large number of mathematical operations simultaneously. Accessing and transferring data is by comparison significantly more expensive. Moving data from main memory into cache, and data transfer between compute nodes can affect performance. The ability to partition the problem into independent parts is becoming more important than minimizing the overall number of mathematical operations.^26^ In this respect, the multiresolution analysis (MRA) framework has an advantage because of the orthogonality, by construction, of the basis. Similar points have been recently raised by other authors.^27,28^ Algorithms that are designed by “thinking parallel” are well suited to effectively exploit the computational power of modern, massively parallel computing clusters.^29,30^

For low or medium precision, traditional basis set approaches are still faster. It is however fair to say that finite basis set methods benefit from decades of development by thousands of scientists, while only a few groups are currently developing Multiwavelet methods for quantum chemistry calculations. We can therefore expect that the potential for further optimizations of the method and extension of the range of applications will increase widely in the future.

The current work has focused on the HF method. For density functional theory (DFT) methods, it has already been shown that multiresolution analysis (MRA) methods can be competitive with respect to computation time.^2^ The most demanding contribution, namely, the exact exchange, is also present for hybrid density functional theory (DFT) functionals and the present discussion therefore applies for density functional theory (DFT) without substantial modifications. The remaining issues concern correlated methods, which are still an active field of development for multiresolution analysis (MRA) methods.^31−33^

## Supporting Information Available

Short introduction to the multiresolution analysis (MRA) representation of functions using MW bases, detailed discussion of the mathematical operations needed to perform a self-consistent field (SCF) cycle, and the available implementation strategies for each of them (PDF)

## Notes

The MRChem code is available at https://github.com/MRChemSoft/mrchem.^5^ The molecule geometries, main input parameters, and model outputs can be found at https://doi.org/10.18710/96NRIX.^34^

## Footnotes

1 The online interactive chart at https://colin-scott.github.io/personal_website/research/interactive_latency.html (Accessed 2022-10-29) is useful to understand the relative performance of on-chip vs off-chip memory accesses. In particular, it shows how the evolution of memory chips has been lagging behind that of CPUs.

2 A similar development has happened for the LCAO methods: they first were based on MOs, but when the focus shifted toward large systems, it was soon realized that efficient implementations demanded a design based on using atomic orbitals (AOs) directly.^7^
