# Supplementary material for: MRChem Multiresolution Analysis Code for Molecular Electronic Structure Calculations: Performance and Scaling Properties
Source: J Chem Theory Comput. 2022 Nov 21;19(1):137–46. doi: 10.1021/acs.jctc.2c00982 (PMC9835826; doi:10.1021/acs.jctc.2c00982)
Supplement: Supplementary file 1 — ct2c00982_si_001.pdf [file ct2c00982_si_001.pdf]

Supporting Information: The MRChem multiresolution analysis code for molecular electronic structure calculations: performance and scaling properties

Peter Wind,[*,†] Magnar Bjørgve,[†] Anders Brakestad,[†] Gabriel A. Gerez S.,[†] Stig Rune Jensen,[*,†] Roberto Di Remigio Eikås,[‡] and Luca Frediani[*,†]

†*Department of Chemistry, UiT - The Arctic University of Norway, N-9037 Tromsø, Norway*

‡*Algorithmiq Ltd, Kanavakatu 3C, FI-00160 Helsinki, Finland*

E-mail: peter.wind@uit.no; stig.r.jensen@uit.no; luca.frediani@uit.no

A note on this document: equations and sections referenced in the Supporting Information refer to the main manuscript, unless the reference number contains the SI prefix.

SI 1 Function representation with Multiwavelets

In order to make the implementation details clearer to the reader we will here introduce a few simple concepts of Multiresolution Analysis (MRA). We will by no means be exhaustive and refer the interested reader to the rich literature on the subject for further details.[S1–S4] In particular, the 2002 article by Alpert et al. is a very pedagogical introduction to multiwavelets (MWs).[S1]

A one dimensional MRA is obtained by defining a set of scaling functions $\phi_p(x)$, which fulfill the so called self-similarity properties: the $k+1$ scaling functions ($p = 0, 1 \ldots k$) in interval $[l/2^s, (l+1)/2^s]$ can be obtained by translating and dilating the original functions defined in $[0, 1]$:

$$\phi^s_{pl}(x) = 2^{s/2}\phi_p(2^s x - l). \tag{SI 1}$$

The wavelet functions are the orthogonal complement of the scaling functions between two successive scales s and $s+1$ and they also fulfill a self-similarity relationship:

$$\psi^s_{pl}(x) = 2^{s/2}\psi_p(2^s x - l). \tag{SI 2}$$

For details about the construction of the Multiwavelets used in this work we refer the reader to the original work of Alpert.[S5] For our purposes, it suffices to say that this construction provides a basis which is dense in L^2 and there are two equivalent ways to project a function, commonly called *reconstructed* and *compressed* representation. The reconstructed representation of a function f makes use of the scaling functions at the finest scale S (largest s value),

$$f = \sum_{l=0}^{2^S-1} \sum_{p=0}^{k} c^S_{pl}\phi^S_{pl}, \qquad c^s_{pl} = \langle f|\phi^s_{pl}\rangle . \tag{SI 3}$$

The compressed representation makes use of the scaling functions at $s = 0$ and all successive wavelet functions from scale $s = 0$ to scale $s = S - 1$.

$$f = \sum_{p=0}^{k} c_p\phi_p + \sum_{s=0}^{S-1} \sum_{l=0}^{2^s-1} \sum_{p=0}^{k} d^s_{pl}\psi^s_{pl}, \qquad d^s_{pl} = \langle f|\psi^s_{pl}\rangle \tag{SI 4}$$

In practice, the *reconstructed* representation describes the function as a piece-wise polynomial on a grid. Such a grid is generally *adaptive* and the finest scale S is therefore not a uniform value. The *compressed* representation is instead used to determine the level of refinement which is necessary to keep the error under control. Both function representations can be expressed as a tree structure where each pair of indices sl designates a MW node in the tree.

The range of l is both scale- and function-dependent: it runs over all nodes included at the given scale for the selected function/orbital. For the reconstructed representation the nodes in use are the *leaf* nodes in the tree (nodes with no further ramifications), whereas for the compressed representation, the nodes in use are all the *branch* nodes, starting from the single root node ($s = 0$). Switching from one representation to the other is performed through the fast MW transform.[S2,S3] For the purpose of the present discussion, suffices to say that the MW transform is local and fast: the computational cost of the MW transform scales as the number of nodes in the function tree and requires only a few small matrix multiplications for each node.

For operator applications in the non-standard form,[S2,S3] both scaling and wavelet coefficients are needed and they are therefore stored for each MW node. As a result, each three dimensional node will contain some metadata (scale s, translation indices l_x, l_y, l_z, connectivity information about neighbors nodes, parent and children, etc...) and an array of $2^3(k+1)^3$ coefficients: $(k+1)^3$ scaling coefficients and $(2^3-1)(k+1)^3$ wavelet coefficients.

SI 2 The basic operations in the SCF iterations

We will here list the main operations involved in the Self-Consistent Field (SCF) procedure, with emphasis on those aspects which are relevant for an efficient parallelization. For a detailed description of the SCF optimization using MWs, the reader is referred to a recently published article which describes the algorithm in detail.[S6]

The starting point is an initial set of orthonormal orbitals which define a Slater determinant for the given problem. A density is computed and the corresponding operators (J and K) are built. The Fock matrix is computed as in Eq. (2), which requires the application of the kinetic operator (T), nuclear potential (V_{nuc}), Coulomb (J) and exchange (K) operators on each occupied orbital. The argument for the application of the integral operator is assembled (the square bracket in Eq. (3)). Then the integral operator is applied separately for

each orbital and the cycle is iterated after an orthonormalization step. Algorithm 1 depicts these main steps.

The elementary mathematical operations required in each SCF iteration are:

- Projection of functions in a MW representation, given their analytic form or a representation on an arbitrary grid.[a]
- Evaluation of the MW representation of functions at a given point in space.
- Multiplication of a function by a number.
- Sum of two MW functions.
- Product of two MW functions.
- Computation of the overlap integral between two MW functions.
- Application of a multiplicative operator, *e.g.* a potential, on a MW function.
- Evaluation of the first order derivative of a MW function.
- Construction of the separated representation of the Poisson and Helmholtz kernels as MW functions.
- Application of the Poisson or Helmholtz operators on a MW function.

All these operations are provided by the MRCPP library.[S7] They must be composed to achieve a SCF implementation, and they need to be integrated in a parallel framework to achieve good scaling and performance. In addition, the SCF cycle requires schemes for orbital orthonormalization, rotation, and localization, Fock matrix diagonalization, history storage and utilization to enable convergence acceleration, using *e.g.* the Krylov-accelerated inexact Newton (KAIN) method.[S8]

[a] As long as a function is *algorithmically* known, that is, there exists an implementation that given a point in the domain, for example $\mathbb{R}^3$, returns the function value, then it is possible to obtain its MW representation.

Algorithm 1 Pseudocode for a single self-consistent field iteration. Each step takes advantage of adaptivity and screening, as described in Sections 3.2 and 3.3.

procedure SCF ITERATION($\{\varphi_i^{[n]}\}_{i=1}^{N_{\mathrm{occ}}}$, ϵ)

Build Coulomb operator:

$$J^{[n]} = \sum_i \left[\frac{1}{|\boldsymbol{r} - \boldsymbol{r}'|} \star \left(\varphi_i^{*,[n]} \varphi_i^{[n]} \right) \right]$$

▷ See Section SI 2.3

Build and apply Exchange operator:

$$K^{[n]} \varphi_j^{[n]} = \sum_i \varphi_i^{[n]} \left[\frac{1}{|\boldsymbol{r} - \boldsymbol{r}'|} \star \left(\varphi_i^{*,[n]} \varphi_j^{[n]} \right) \right]$$

▷ See Section SI 2.4

Assemble Fock matrix elements:

$$F_{ij}^{[n]} = \left\langle \varphi_i^{[n]} \middle| T + V_{\mathrm{nuc}} + J^{[n]} - K^{[n]} \middle| \varphi_j^{[n]} \right\rangle$$

▷ See Sections SI 2.1 and SI 2.2

Assemble right-hand side:

$$V^{[n]} \varphi_i^{[n]} - \sum_{j \neq i}^{N_{\mathrm{occ}}} F_{ij}^{[n]} \varphi_j^{[n]}$$

▷ See Section SI 2.5

Update orbitals:

$$\varphi_i^{[n+1]} = -2 H^{\mu_i} \star \left[V^{[n]} \varphi_i^{[n]} - \sum_{j \neq i}^{N_{\mathrm{occ}}} F_{ij}^{[n]} \varphi_j^{[n]} \right]$$

▷ See Section SI 2.5

Accelerator and localization: A new set of orbitals is created using results from previous iterations and localization. This step is optional and does not need to be performed at each iteration.

end procedure

SI 2.1 The kinetic operator T

The application of the kinetic energy operator on a MW function is formally not well defined due to the discontinuities in the basis.[S9] However, when adopting an integral reformulation of the SCF equations, the kinetic energy operator is required only in the expectation value expressions as in Eq. (2), and as shown by Anderson et al.[S9] the original definition of the first-order derivative by Alpert et al.[S1] is able to deliver sufficient precision in this specific case. On the basis of this consideration, and for computational efficiency, the matrix elements of the Laplacian operator are computed as inner products of orbital gradients:

$$T_{ij} = -\frac{1}{2} \left\langle \varphi_i \,\middle|\, \nabla^2 \varphi_j \right\rangle = \frac{1}{2} \left\langle \nabla \varphi_i \,\middle|\, \nabla \varphi_j \right\rangle . \qquad \text{(SI 5)}$$

It is possible to avoid the kinetic energy operator altogether,[S6] but in practice this complicates the algorithm to the point where the direct computation as in Eq. (SI 5) is to be preferred for large systems.

SI 2.2 The nuclear potential V_{nuc}

The nuclear potential consists of a sum of contributions from the different nuclei. As such, applying it to a given orbital can be performed in two ways.

apply-then-sum The potential from each nucleus α is applied to each orbital and the results summed up over all nuclei:

$$V_{\text{nuc}} \varphi_j = \sum_{\alpha}^{N_{\text{atoms}}} \left(V_\alpha \varphi_j \right), \qquad \text{(SI 6)}$$

sum-then-apply The sum over the nuclei is performed first, followed by the multiplication with the given orbital:

$$V_{\text{nuc}} \varphi_j = \left(\sum_{\alpha}^{N_{\text{atoms}}} V_\alpha \right) \varphi_j . \qquad \text{(SI 7)}$$

The first approach requires $N_{\text{atoms}} \times N_{\text{occ}}$ function multiplications, followed by a summation of N_{atoms} terms. It also needs memory for $N_{\text{atoms}} \times N_{\text{occ}}$ MW functions. However, since these objects are relatively localized, their memory footprint is modest and can, in addition, be amortized by distributing objects across parallel processes. Furthermore, the function-function products can also be distributed across nodes and screened aggressively.

The second approach requires a summation of N_{atoms} terms, followed by N_{occ} function multiplications. However, the total potential is no longer well-localized, as it describes the potential from all clamped nuclei, and it requires a large amount of memory storage. Storing the nuclear potential is wasteful: only its MW nodes with non-zero overlap with the occupied orbitals will contribute to the final result.

Despite these considerations, our present implementation uses the "sum-then-apply" form. In the future we plan to try the first approach and also adapt the accuracy according to the electronic density and compare the performance between the two approaches.

SI 2.3 The Coulomb potential J

The Coulomb interaction can be expressed as:

$$J(\boldsymbol{r}) = \sum_i \int \frac{\varphi_i^*(\boldsymbol{r}')\varphi_i(\boldsymbol{r}')}{|\boldsymbol{r}-\boldsymbol{r}'|}\,\mathrm{d}\boldsymbol{r}' = \sum_i \left[\frac{1}{|\boldsymbol{r}-\boldsymbol{r}'|} \star \varphi_i^*\varphi_i\right] = \frac{1}{|\boldsymbol{r}-\boldsymbol{r}'|} \star \left[\sum_i \varphi_i^*\varphi_i\right] \tag{SI 8}$$

where, as indicated by the last step, we can choose the order of the operations when constructing the Coulomb potential: either summing the orbital densities first and compute the potential of the total density (**sum-then-apply**), or, first computing the Coulomb potential of each individual orbital density and then sum the potentials (**apply-then-sum**). The electrostatic potential is in general a much smoother function than its corresponding density, which contains cusps at every nuclear site. It is thus desirable to avoid the explicit construction of the global density and instead gradually build a global potential by partial applications of the Poisson operator to subsets of orbital densities. This operation can also

easily be distributed in parallel by assigning different subsets to different processes.

The Poisson kernel is expressed in a separable form, within the requested precision, as a sum of Gaussian contributions.[S10] Each Gaussian kernel is applied separately and the contributions are then added up. The separated representation is essential to achieve fast linear-scaling algorithms: on the one hand it brings down the formal scaling of the operator application for each node from 2^6k^6 to $M2^4k^4$, where k is the polynomial order and M is the separation rank; on the other hand the Gaussian contributions will range from wide ones (shallow MW representation which couple long-range but at coarse scales) to narrow ones (deep MW representation which couple short-range at all scales). In practice, most of the M terms will be applied very efficiently and the separation between long-range and short-range interactions – which is otherwise achieved through the fast multipole method (FMM) – is naturally included without extra effort.[S2,S11]

Normally the potential is computed with the required precision in the entire space, and since it is long range, it will be relatively memory consuming.

SI 2.4 The exchange potential K

The construction and application of the exchange potential is the most computationally demanding part, because the exchange operator cannot be written as a simple multiplicative potential. A Coulomb potential must be constructed and applied for each orbital pair:

$$K[\varphi_j] = \sum_i K_i[\varphi_j] = \sum_i \varphi_i(\boldsymbol{r}) \int \frac{\varphi_i^*(\boldsymbol{r}')\varphi_j(\boldsymbol{r}')}{|\boldsymbol{r}-\boldsymbol{r}'|}\,\mathrm{d}\boldsymbol{r}' = \sum_i \varphi_i(\boldsymbol{r}) \left[\frac{1}{|\boldsymbol{r}-\boldsymbol{r}'|} \star \varphi_i^*\varphi_j\right] \quad \text{(SI 9)}$$

Section 3.4 provides a more detailed description of how these terms can be evaluated efficiently in a MRA framework.

SI 2.5 Construction of the right-hand side and Helmholtz operator application

The central step in the SCF iteration is the application of the Helmholtz operator H^{μ} defined in Eq. (4) to the square bracket on the right-hand side in Eq. (3). The Helmholtz kernel depends on the orbital energy ($\mu_i = \sqrt{-2F_{ii}}$). As such, one needs to construct as many operators as there are occupied orbitals. Given the Fock matrix, each term within the square bracket in Eq. (3) can be assembled with $O(N)$ formal operation count per orbital φ_i. Thus, the formation of the whole right-hand side for the entire set of orbitals formally scales as $O(N^2)$. However, $\sum_{j\neq i}^{N_{\mathrm{occ}}} F_{ij}\varphi_j$ is a linear combination of orbitals and, as shown in Section 3.2, it can be performed efficiently with $O(N)$ operation count. Also the application of the exchange operator can be done essentially in $O(N)$ operations as shown in Sections 3.4 and 4.4.

Once the right-hand side is assembled, the application of the Helmholtz operator is practically identical to the Coulomb operator. The only difference is the actual expansion in Gaussians, which, thanks to the long-range exponential decay of the kernel, has more favorable scaling properties.[S2]

References

(S1) Alpert, B.; Beylkin, G.; Gines, D.; Vozovoi, L. Adaptive Solution of Partial Differential Equations in Multiwavelet Bases. *Journal Of Computational Physics* **2002**, *182*, 149–190.

(S2) Frediani, L.; Fossgaard, E.; Flå, T.; Ruud, K. Fully adaptive algorithms for multivariate integral equations using the non-standard form and multiwavelets with applications to the Poisson and bound-state Helmholtz kernels in three dimensions. *Molecular Physics* **2013**, *111*, 1143–1160.

(S3) Beylkin, G.; Coifman, R.; Rokhlin, V. Fast wavelet transforms and numerical algorithms I. *Communications on Pure and Applied Mathematics* **1991**, *44*, 141–183.

(S4) Bischoff, F. A. In *Advances in Quantum Chemistry*; Ancarani, L. U., Hoggan, P. E., Eds.; Academic Press, 2019; Vol. 79; pp 3–52.

(S5) Alpert, B. K. A Class of Bases in L^2 for the Sparse Representation of Integral Operators. *SIAM Journal on Mathematical Analysis* **1993**, *24*, 246–262.

(S6) Jensen, S. R.; Durdek, A.; Bjørgve, M.; Wind, P.; Flå, T.; Frediani, L. Kinetic energy-free Hartree–Fock equations: an integral formulation. *Journal Of Mathematical Chemistry* **2022**, 1–19.

(S7) Bast, R.; Bjorgve, M.; Di Remigio, R.; Durdek, A.; Frediani, L.; Fossgaard, E.; Gerez, G.; Jensen, S. R.; Juselius, J.; Monstad, R.; Wind, P. MRCPP: MultiResolution Computation Program Package. 2021; `https://doi.org/10.5281/zenodo.5566943`, Accessed: 2022-10-29.

(S8) Harrison, R. J. Krylov subspace accelerated inexact Newton method for linear and nonlinear equations. *Journal of Computational Chemistry* **2004**, *25*, 328–334.

(S9) Anderson, J.; Harrison, R. J.; Sekino, H.; Sundahl, B.; Beylkin, G.; Fann, G. I.; Jensen, S. R.; Sagert, I. On derivatives of smooth functions represented in multiwavelet bases. *Journal of Computational Physics: X* **2019**, *4*, 100033.

(S10) Beylkin, G.; Monzón, L. On approximation of functions by exponential sums. *Applied and Computational Harmonic Analysis* **2005**, *19*, 17–48.

(S11) Beylkin, G.; Cramer, R.; Fann, G.; Harrison, R. J. Multiresolution separated representations of singular and weakly singular operators. *Applied and Computational Harmonic Analysis* **2007**, *23*, 235–253.
